# Prevalence and types of headache, sleep disturbances and hypertension among non-psychotic patients

**DOI:** 10.1186/s12883-025-04579-6

**Published:** 2025-12-09

**Authors:** Ahmad Neyazi, Mehrab Neyazi, Mehran Neyazi, Nosaibah Razaqi, Abdul Qadim Mohammadi

**Affiliations:** 1 Afghanistan Center for Epidemiological Studies, Herat, Afghanistan; 2 Scientific Research Committee, Afghanistan Medical Students Association, Herat, Afghanistan; 3 Department of Mental Health, Herat Regional Hospital, Herat, Afghanistan

**Keywords:** Sleep quality, Hypertension, Headache disorders, Psychosocial stress, Afghanistan

## Abstract

**Background:**

Headaches, sleep disturbances, and hypertension are common and interrelated conditions that significantly affect quality of life. This study aimed to assess the prevalence and sociodemographic predictors of these conditions among non-psychotic patients in Afghanistan.

**Methods:**

A prospective cross-sectional survey was conducted from September 5, 2024, to September 1, 2025, in Herat province (*N* = 875). Sleep disturbances were assessed using the Pittsburgh Sleep Quality Index (PSQI), with scores ≥ 5 indicating poor sleep quality. Hypertension was defined as systolic blood pressure ≥ 140 mmHg and/or diastolic blood pressure ≥ 90 mmHg. Headache was defined as the presence of any clinically diagnosed headache type. Multivariable logistic regression models were used to identify independent predictors of sleep disturbances, hypertension, and headache.

**Results:**

Poor sleep quality was reported by 72.7% of participants, and 54.7% met criteria for hypertension. Female sex, widowhood, and rural residency predicted poor sleep quality. Older age (≥ 36 years), widowhood, smoking, and sleep disturbance were independent predictors of hypertension. Female sex and widowhood were significant predictors of headache, with widowed individuals showing the highest odds across all three conditions.

**Conclusion:**

Sleep disturbances, hypertension, and headaches were highly prevalent in this Afghan clinical population. Older age, female gender, widowhood, and smoking emerged as key predictors, with widowhood representing the strongest vulnerability factor. Targeted interventions addressing psychosocial stress, sleep quality, and cardiovascular risk in socioeconomically disadvantaged groups—particularly widows—are urgently needed.

## Introduction

Headaches, sleep disturbances, and hypertension represent significant global health challenges that profoundly impact an individual’s quality of life and are frequently observed as interconnected conditions [1–3]. While the prevalence and mechanisms of each condition have been extensively investigated individually [4, 5], their intricate interrelationships, particularly within specific clinical populations such as non-psychotic patients in regions like Afghanistan, remain less explored [6]. The World Health Organization (WHO) has recognized hypertension as a leading risk factor for disability-adjusted life years (DALYs) globally, contributing to 7.5 million deaths annually [1, 7]. In Afghanistan, for instance, the prevalence of hypertension symptoms was reported at 20.9% among hospitalized patients, alongside a high prevalence of depressive symptoms at 65.8% [1]. Furthermore, hypertension has been linked to compromised health-related quality of life among various populations, including school teachers in Afghanistan [8].

Sleep is a fundamental physiological process crucial for physical and mental restoration [2, 9]. Disruptions in sleep quality are widespread and have serious detrimental effects on overall health, affecting an individual’s physical and social functioning, professional potential, and general quality of life [2]. Studies have consistently indicated a close relationship between poor sleep quality and chronic diseases, including hypertension and cardiovascular diseases [10]. The Pittsburgh Sleep Quality Index (PSQI) is a widely utilized tool for assessing sleep quality over a one-month period [11–13]. A PSQI score of 5 or more often indicates poor sleep quality [14]. Poor sleep quality has been linked to various conditions, such as pulmonary arterial hypertension (PAH) [15], idiopathic intracranial hypertension [5, 11], and chronic liver disease [16]. In stroke survivors and caregivers, poor sleep quality is associated with increased depressive symptoms [17]. Among Afghan healthcare workers and female school students, sleep quality is influenced by various sociodemographic factors and anxiety levels, respectively [6, 18].

The bidirectional relationship between sleep quality and hypertension is particularly notable [2]. Poor sleep can exacerbate hypertensive symptoms, and conversely, the degree of hypertension can directly affect sleep quality [2]. Research suggests that individuals with sleep disorders are at an increased risk for cognitive function decline, especially in hypertensive patients [19]. A decision-tree analysis reveals that physical activity level, type of work, and sleep disorders are significant factors associated with the presence of hypertension, with those having sleep disorders showing a higher probability of hypertension [19].

Headaches, including primary headache types like migraine and tension-type headache, are prevalent neurological conditions that can impair quality of life [4, 20]. Primary headache is also a common comorbidity in patients with epilepsy, affecting their health-related quality of life [20]. Headaches can also be a symptom of underlying conditions such as idiopathic intracranial hypertension [5, 11] or Behcet’s syndrome [21]. Studies have explored the spectrum of headache disorders in patients with patent foramen ovale (PFO), extending beyond the known migraine-PFO connection [4]. The interplay between headaches, sleep disturbances, and hypertension forms a complex clinical picture, as sleep disturbances can exacerbate the frequency and intensity of headaches [22] while hypertension itself may contribute to headache pathology. For instance, in a study assessing therapeutic horticulture, anxiety subjects showed significant differences in total PSQI, sleep latency, sleep disturbances, and daytime dysfunction compared to non-anxiety subjects, particularly on day 1, indicating the intricate link between anxiety, sleep, and potentially headache experience [22].

Despite the growing body of literature on these individual conditions, a comprehensive understanding of their co-occurrence and contributing factors within specific patient populations in under-researched geographical areas is crucial [6]. The socio-cultural and healthcare landscape of Afghanistan presents unique challenges and warrants specific investigation into these health concerns. Prior research in Afghanistan has highlighted the prevalence of sleep disturbances and their association with quality of life among adults [23], as well as anxiety symptoms and sleep disturbance among female school students [18]. However, few studies have systematically evaluated the multifaceted interplay of headache types, sleep disorders, and hypertension in non-psychotic patients within a clinical setting in this region. To address this gap, the present study focuses specifically on the triad of poor sleep quality (PSQI ≥ 5), hypertension, and clinically classified headache types among Afghan non-psychotic patients. This study aims to determine their prevalence and to identify the sociodemographic predictors associated with each condition. Based on existing evidence, we hypothesized that older age, female gender, and widowhood would be associated with a higher likelihood of experiencing poor sleep quality, hypertension, and headaches.

## Materials and methods

### Participants, study design, and procedure

A cross-sectional study was conducted, involving 875 non-psychotic patients (205 men and 670 women). Data collection was carried out through face-to-face interviews conducted by trained data collectors. Data were collected prospectively between September 5, 2024, and September 1, 2025. Recruitment occurred in the outpatient psychiatry clinic and the internal medicine outpatient department of Herat Regional Hospital. Data collectors were present during morning and afternoon shifts on all weekdays, ensuring coverage of routine clinical operations. Weekend clinics were not active; therefore, data were not collected on weekends. Eligibility criteria included: (i) residence in Afghanistan, (ii) a minimum age of 18 years, (iii) proficiency in understanding either Dari and/or Pashto, and (iv) the ability to provide informed consent, either written or verbal.

### Instruments

The study utilized a survey consisting of five sections. The first section collected socio-demographic data, the second addressed sleep disturbances, the third focused on non-psychotic diseases, the fourth assessed hypertension, and the fifth evaluated types of headaches among participants. The socio-demographic section included questions on age, gender, marital status, residence, education level, economic status (classified based on monthly family income as either greater than or less than $100), and cigarette smoking status (categorized as never smoked, ex-smoker, or current smoker).

PSQI was employed to evaluate sleep quality. The instrument consists of 19 self-rated items and six optional items rated by a bed partner or roommate; however, only the self-rated items were included in the scoring. The 19 items are organized into seven components: subjective sleep quality, sleep latency, sleep duration, habitual sleep efficiency, sleep disturbances, use of sleep medication, and daytime dysfunction. Each item (e.g., “Difficulty falling asleep within 30 minutes”) is scored on a scale from 0 (“not during the past month”) to 3 (“three or more times per week”). The global score, ranging from 0 (indicating no difficulty) to 21 (indicating severe difficulty across all components), is obtained by summing the seven component scores. In this study, a total PSQI score of < 5 was classified as good sleep quality, while a score ≥ 5 indicated poor sleep quality. Internal consistency for the scale in the current sample was acceptable, with a Cronbach’s alpha of 0.777.

The assessment of non-psychotic disorders was conducted by psychiatry specialists using structured clinical interviews informed by DSM-5 diagnostic criteria. Specialists performed comprehensive interviews to gather detailed histories, systematically assessing symptom clusters, onset, duration, severity, and functional impairment following DSM-5 guidelines to ensure diagnostic consistency across patients. While full structured diagnostic instruments (e.g., SCID-5) were not used due to resource constraints, clinicians also conducted physical examinations and collaborated with other healthcare providers when necessary to rule out underlying medical conditions or comorbidities. This approach allowed for the precise identification of non-psychotic disorders, forming the basis for evidence-based interventions tailored to the needs of the participants.

Blood pressure was measured for each participant using clinically validated device with an appropriately sized cuff on the right arm, while seated with back supported and feet flat on the floor. After a 5-minute rest, the first reading was taken, followed by a second reading after a 2-minute interval. The mean of the two readings was used for analysis. Hypertension was defined as a systolic pressure ≥ 140 mmHg and/or diastolic pressure ≥ 90 mmHg, or self-reported current use of antihypertensive medication, regardless of measured values.

Headache types were classified according to the International Classification of Headache Disorders, 3rd edition (ICHD-3) diagnostic principles, with a final categorization made by psychiatry specialists into migraine, tension-type, cluster, post-traumatic, or unspecified headache. This assessment was based on a clinical evaluation during which detailed medical histories were obtained, focusing on the onset, frequency, duration, intensity, and associated symptoms of the headaches. While this approach reflects routine clinical practice in the study setting, the absence of a structured diagnostic questionnaire or a formal ICHD-3 checklist may limit replicability.

### Analysis

A minimum sample size was estimated based on logistic regression requirements of at least 10–15 events per predictor variable. With 636 cases of poor sleep quality and 479 cases of hypertension, the sample provided sufficient statistical power to support the multivariable models. Data entry was performed using Microsoft Excel 2016, and analysis was conducted with IBM SPSS version 27.0 for Windows. Descriptive statistics included means, standard deviations, frequencies, and percentages. Associations between variables were assessed using chi-square tests. Multiple regression analysis was applied to identify independent socio-demographic factors associated with sleep disturbances and types of headaches. A two-tailed p-value of less than 0.05 was considered indicative of statistical significance.

## Results

The sample consists of 875 non-psychotic patients, with nearly equal representation across age groups, as 50.1% are under 36 years old and 49.9% are 36 years or older. The majority of participants are female (76.6%) compared to male (23.4%). Marital status shows that most participants are married (78.6%), followed by single individuals (15.4%) and a small proportion who are widowed or divorced (5.8%). In terms of residency, 51.7% live in rural areas, while 48.3% reside in urban areas. Regarding education, the majority are illiterate (74.3%), with smaller percentages having attended school (15.2%) or completed university (10.5%). A significant portion of the sample (93.1%) reports an economic status of earning less than $100, while only 6.9% earn more than $100. The majority of participants are non-smokers (90.9%), with ex-smokers (7.2%) and current smokers (1.9%) being less prevalent. Regarding health, 72.7% of the sample reports sleep disturbances, and 54.7% are hypertensive, while 45.3% have normal blood pressure. **[**Table 1**]**

**Table 1:** Characteristics distribution of the study sample (Afghanistan-2024)

Characteristic	Categories	Total	
		*N*	%
Age group	< 36-years-old >=36-years-old	438 437	50.1 49.9
Gender	Male Female	205 670	23.4 76.6
Marital status	Single Married Widow/divorced	135 689 51	15.4 78.6 5.8
Residency	Urban Rural	423 452	48.3 51.7
Education	Illiterate School University	650 133 92	74.3 15.2 10.5
Economic status	Less than $100 More than $100	815 60	93.1 6.9
Cigarette smoking	Never Ex-smoking Smoker	795 63 17	90.9 7.2 1.9
Sleep disturbances	Absent Present	239 636	27.3 72.7
Blood pressure	Normotensive Hypertensive	396 479	45.3 54.7
Total		**875**	**100.0**

**Fig. 1:**
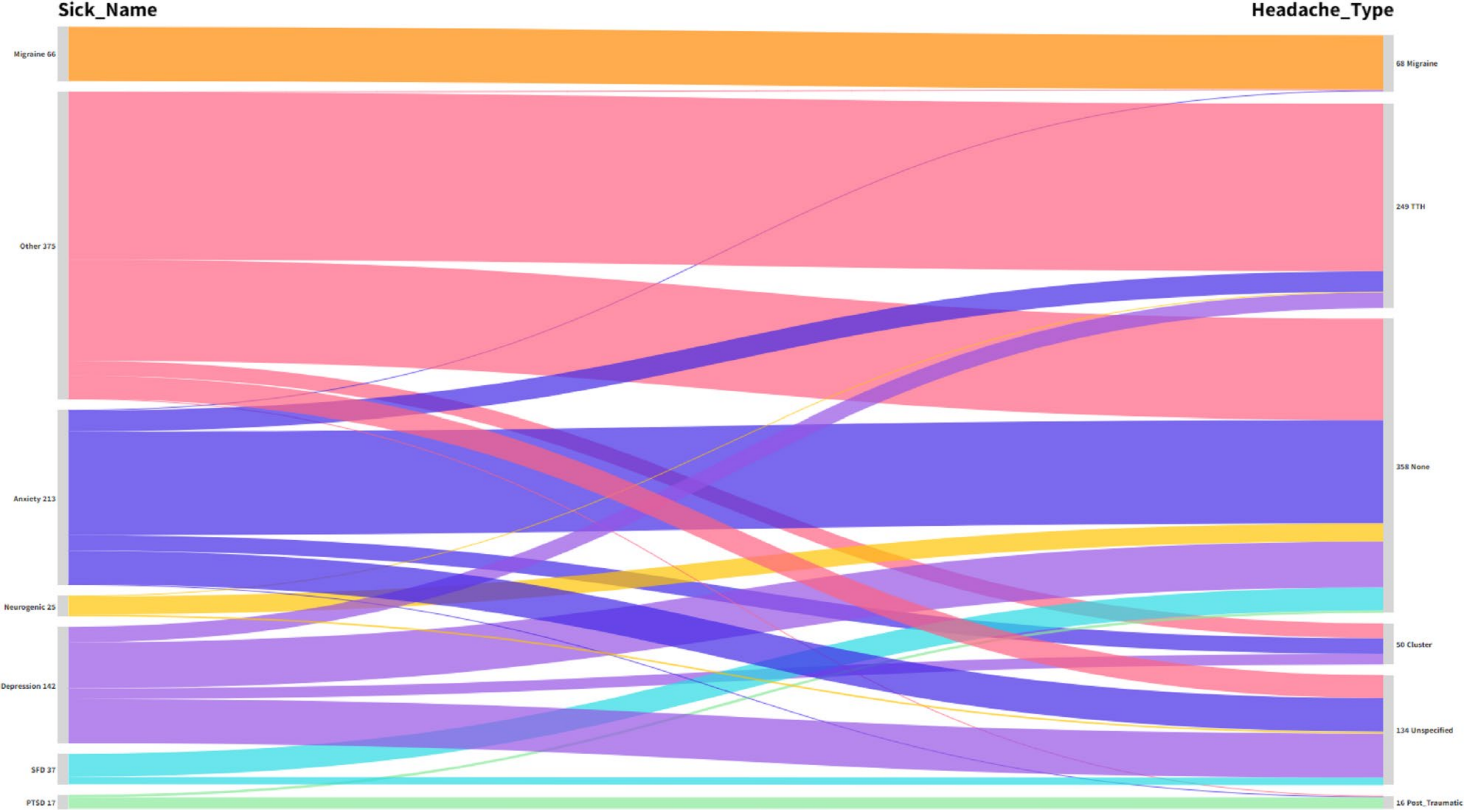
Non-psychotic disorder and their associated types of headaches (Afghanistan 2024)

Migraine (66 non-psychotic patients) is exclusively associated with Migraine headaches (100%). Anxiety (213 individuals) is predominantly linked to the None category (59.2%), indicating that a majority of individuals with anxiety do not report headaches, while smaller proportions experience Tension (11.7%) or Cluster headaches (8.9%). Similarly, Depression (142 individuals) shows a strong association with None (39.4%) and Tension (13.4%), with no cases of Migraine headaches. Neurogenic disorders (25 individuals) are mostly associated with None (88.0%), with minor reports of Tension (4.0%). PTSD (375 individuals) is primarily linked to post-traumatic headaches (82.4%) and None (17.6%). SFD (17 individuals) shows a significant association with None (75.7%) and a small percentage reporting Unspecified headache (24.3%). The Other category (37 non-psychotic patients) is predominantly associated with Tension (54.4%) and None (32.8%), with very few reporting Migraine. **[**Figure 1**] [**Table 2**]**.

**Table 2:** Non-psychotic disorders and their types of headaches

Disorder	Cluster (%)	Migraine (%)	None (%)	Post Traumatic (%)	Tension (%)	Unspecified (%)	Total
Anxiety	19a (8.9)	1b (0.5)	126a (59.2)	1a, b (0.5)	25b (11.7)	41a (19.2)	**213 (24.3)**
Depression	13a, b (9.2)	0c (0.0)	56b (39.4)	0b, c (0.0)	19c (13.4)	54a (38.0)	**142 (16.2)**
Migraine	0a (0.0)	66b (100.0)	0a (0.0)	0a (0.0)	0a (0.0)	0a (0.0)	**66 (7.5)**
Neurogenic	0ab (0.0)	0a, b (0.0)	22b (88.0)	0a, b (0.0)	1a (4.0)	2a, b (8.0)	**25 (2.9)**
PTSD	0a (0.0)	0a (0.0)	3a (17.6)	14b (82.4)	0a (0.0)	0a (0.0)	**375 (42.9)**
SFD	0a, b,c (0.0)	0a, b,c (0.0)	28c (75.7)	0a, b,c (0.0)	0b (0.0)	9a, c (24.3)	**17 (1.9)**
Other	18a (4.8)	1b (0.3)	123a (32.8)	1a, b (0.3)	204c (54.4)	28a (7.5)	**37 (4.2)**
Total	**50 (5.7)**	**68 (7.8)**	**358 (40.9)**	**16 (1.8)**	**249 (28.5)**	**134 (15.3)**	**875 (100.0)**

**Fig. 2:**
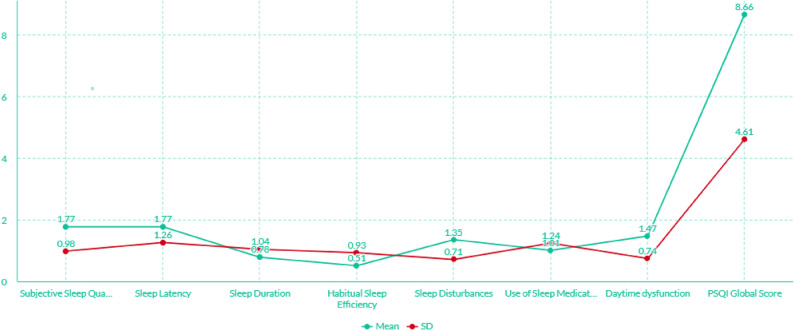
Sleep disturbances components among participants in this study (Afghanistan 2024)

Figure 2 illustrates the mean component score profiles of the study participants as assessed by the PSQI. The mean scores for subjective sleep quality, sleep duration, sleep disturbances, and use of sleep medication were 1.77, 0.78, 1.35, and 1.01, respectively. The mean global PSQI score was 8.66, indicating poor sleep quality among the participants. Among the PSQI components, subjective sleep quality had the highest mean score (1.77), indicating that participants most frequently perceived their sleep as poor. This aligns with the elevated global PSQI score (8.66) and suggests that the most pronounced aspect of sleep disturbance in this population is the subjective experience of inadequate or unsatisfying sleep, which may reflect underlying psychosocial stressors or environmental challenges. **[**Figure 2**]**

Age group is strongly associated with sleep disturbances, as non-psychotic patients aged 36 years or older report a higher prevalence of sleep disturbances (79.9%, *p* < 0.001). Gender also plays a role, with females experiencing a higher rate of sleep disturbances compared to males (75.7% vs. 62.9%, *p* < 0.001). Marital status is another significant factor, with married and widowed/divorced individuals reporting higher rates of sleep disturbances (77.5% and 88.2%, respectively, *p* < 0.001). While residency (urban vs. rural) shows a weaker association (*p* = 0.059), education level is highly significant, with illiterate individuals having the highest rate of sleep disturbances (76.2%, *p* < 0.001). Economic status shows a trend toward a higher rate of disturbances among those earning more than $100 (83.3%, *p* = 0.055), although this is not statistically significant. Cigarette smoking status does not appear to affect the prevalence of sleep disturbances (*p* = 0.415). Finally, blood pressure is strongly associated with sleep disturbances, as individuals with hypertension report a higher prevalence (78.3%, *p* < 0.001). Overall, sleep disturbances are more common among older individuals, females, those with lower education levels, hypertensive patients, and those who are married or widowed/divorced. **[**Table 3**]**

**Table 3:** Association of sleep disturbances with participants socio-demographic characteristics (Herat, Afghanistan-2024)

Characteristic	Categories	Sleep disturbances		*p*-value
		Absent	Present	
		*N* (%)	*N* (%)	
Age group	< 36-years-old >=36-years-old	151 (34.5) 88 (20.1)	287 (65.5) 349 (79.9)	**< 0.001**
Gender	Male Female	76 (37.1) 163 (24.3)	129 (62.9) 507 (75.7)	**< 0.001**
Marital status	Single Married Widow/divorced	78 (57.8) 155 (22.5) 6 (11.8)	57 (42.2) 534 (77.5) 45 (88.2)	**< 0.001**
Residency	Urban Rural	128 (30.3) 111 (24.6)	295 (69.7) 341 (75.4)	0.059
Education	Illiterate School University	155 (23.8) 42 (31.6) 42 (45.7)	495 (76.2) 91 (68.4) 50 (54.3)	**< 0.001**
Economic status	Less than $100 More than $100	229 (28.1) 10 (16.7)	586 (71.9) 50 (83.3)	0.055
Cigarette smoking	Never Ex-smoking Smoker	214 (26.9) 18 (28.6) 7 (41.2)	581 (73.1) 45 (71.4) 10 (58.8)	0.415
Blood pressure	Normotensive Hypertensive	135 (34.1) 104 (21.7)	261 (65.9) 375 (78.3)	**< 0.001**
Total		**239 (27.3)**	**636 (72.7)**	

Age is strongly associated with blood pressure, as non-psychotic patients aged 36 years or older have a higher prevalence of hypertension (76.0%, *p* < 0.001). Marital status also shows a significant relationship, with married and widowed/divorced individuals experiencing higher rates of hypertension (61.7% and 80.4%, respectively, *p* < 0.001). Residency is another factor, with rural residents exhibiting a higher rate of hypertension (59.3% vs. 49.9% in urban areas, *p* = 0.005). Education level plays a critical role, as illiterate individuals have the highest proportion of hypertension (62.0%, *p* < 0.001). Cigarette smoking is strongly associated with hypertension, with smokers and ex-smokers showing higher rates of hypertension (82.5% and 29.4%, respectively, *p* < 0.001). Sleep disturbances are also linked to blood pressure status, with individuals experiencing sleep disturbances having a higher prevalence of hypertension (59.0%, *p* < 0.001). Gender and economic status, however, do not show significant associations with blood pressure (*p* = 0.605 and *p* = 0.563, respectively). Overall, hypertension is more prevalent among older non-psychotic patients, those with lower education, rural residents, smokers, and those experiencing sleep disturbances. **[**Table 4**]**

**Table 4:** Association of blood pressure with participants socio-demographic characteristics (Herat, Afghanistan-2024)

Characteristic	Categories	Blood pressure		*p*-value
		Normotensive	Hypertensive	
		*N* (%)	*N* (%)	
Age group	< 36-years-old >=36-years-old	291 (66.4) 105 (24.0)	147 (33.6) 332 (76.0)	**< 0.001**
Gender	Male Female	96 (46.8) 300 (44.8)	109 (53.2) 370 (55.2)	0.605
Marital status	Single Married Widow/divorced	122 (90.4) 264 (38.3) 10 (19.6)	13 (9.6) 425 (61.7) 41 (80.4)	**< 0.001**
Residency	Urban Rural	212 (50.1) 184 (40.7)	211 (49.9) 268 (59.3)	**0.005**
Education	Illiterate School University	247 (38.0) 81 (60.9) 68 (73.9)	403 (62.0) 52 (39.1) 24 (26.1)	**< 0.001**
Economic status	Less than $100 More than $100	371 (45.5) 25 (41.7)	444 (54.5) 35 (58.3)	0.563
Cigarette smoking	Never Ex-smoking Smoker	373 (46.9) 11 (17.5) 12 (70.6)	422 (53.1) 52 (82.5) 5 (29.4)	**< 0.001**
Sleep disturbances	Absent Present	135 (56.5) 261 (41.0)	104 (43.5) 375 (59.0)	**< 0.001**
Total		**396 (45.3)**	**479 (54.7)**	

The findings reveal that age is not associated with sleep disturbances (AOR = 1.209, *p* = 0.304) or headaches (AOR = 0.856, *p* = 0.334), but individuals aged 36 years or older are more likely to have hypertension (AOR = 3.680, *p* < 0.001). Gender is associated with sleep disturbances (females have higher odds, AOR = 1.614, *p* = 0.014) and headaches (females have higher odds, AOR = 1.546, *p* = 0.014), but not with hypertension (AOR = 0.703, *p* = 0.096). Marital status reveals that widows have higher odds of experiencing sleep disturbances (AOR = 6.823, *p* < 0.001), hypertension (AOR = 12.631, *p* < 0.001), and headaches (AOR = 4.491, *p* < 0.001), compared to singles. Residency is weakly associated with sleep disturbances (rural areas have higher odds, AOR = 1.343, *p* = 0.089) but not with hypertension or headaches. Education level shows no significant associations with sleep disturbances, hypertension, or headaches. Economic status is marginally associated with sleep disturbances (AOR = 1.866, *p* = 0.098), but not with hypertension or headaches. These findings suggest that age, gender, marital status, and economic status influence the likelihood of experiencing sleep disturbances, hypertension, and headaches, with particularly strong associations for widows. **[**Table 5**]**

**Table 5:** Multiple logistic regression analysis of presence of sleep disturbances, hypertension, and having headache with participants’ socio-demographic characteristics (Afghanistan 2024)

Variable	Sleep disturbances		Blood Pressure		Headache	
	AOR [95% CI]	*p*-value	AOR [95% CI]	*p*-value	AOR [95% CI]	*p*-value
Age group						
< 36-years-old	Reference		Reference		Reference	
>=36-years-old	1.209 [0.842, 1.737]	0.304	3.680 [2.651, 5.109]	**< 0.001**	0.856 [0.623, 1.174]	0.334
Gender						
*Male*	Reference		Reference		Reference	
*Female*	1.614 [1.104, 2.361]	**0.014**	0.703 [0.465, 1.064]	0.096	1.546 [1.090, 2.193]	**0.014**
Marital status						
*Single*	Reference		Reference		Reference	
*Married*	1.892 [0.779, 4.591]	0.159	1.585 [0.757, 3.319]	0.222	1.319 [0.705, 2.467]	0.387
*Widow/divorced*	6.823 [2.529, 18.407]	**< 0.001**	12.631 [4.779,33.484]	**< 0.001**	4.491 [2.080, 9.697]	**< 0.001**
Residency						
*Urban*	Reference		Reference		Reference	
*Rural*	1.343 [0.956, 1.885]	0.089	1.158 [0.831, 1.614]	0.386	0.981 [0.728, 1.323]	0.900
Education						
*Illiterate*	Reference		Reference		Reference	
*School*	0.720 [0.395, 1.309]	0.281	1.072 [0.542, 2.121]	0.841	0.915 [0.512, 1.634]	0.763
*University*	0.787 [0.461, 1.344]	0.380	0.628 [0.342, 1.153]	0.133	1.272 [0.755, 2.142]	0.367
Economic status						
Less than $100	Reference		Reference		Reference	
More than $100	1.866 [0.892, 3.903]	0.098	0.647 [0.353, 1.188]	0.160	1.346 [0.759, 2.388]	0.310

## Discussion

This study investigated the prevalence and interrelationships of headache types, sleep disturbances, and hypertension among 875 non-psychotic Afghan patients, providing crucial insights into the health burden within this demographic. The findings underscore a high prevalence of sleep disturbances (72.7%) and hypertension (54.7%) within the studied cohort, which aligns with or exceeds rates reported in other populations, particularly for sleep disturbances [1]. For instance, a previous study in Afghanistan found a depression symptom prevalence of 65.8% and hypertension prevalence of 20.9% among hospitalized patients, suggesting variability depending on the specific patient population [1]. The mean global PSQI score of 8.66 indicates generally poor sleep quality in this sample, consistent with findings globally that link poor sleep to a range of adverse health outcomes, including physical, social, and professional functioning impairments [2].

The demographic profile of the study participants, predominantly female (76.6%), married (78.6%), illiterate (74.3%), and with low income (93.1% earning < $100), highlights the vulnerability of this population to health disparities. These socioeconomic factors are often associated with poorer health outcomes and reduced access to healthcare, which could exacerbate the observed high prevalence of chronic conditions [8].

The study revealed distinct associations between headache types and specific non-psychotic disorders. Migraine was exclusively migraine-type among those diagnosed with migraine (*n* = 66), indicating a clear diagnostic classification within this subgroup. A notable finding was the high association of post-traumatic headache (82.4%) with individuals suffering from Post-Traumatic Stress Disorder (PTSD), given that PTSD can often manifest with various somatic symptoms including headaches. The unexpectedly high proportion of patients with anxiety (59.2%) and depression (39.4%) who reported no headache requires careful contextual interpretation. Although anxiety and depressive disorders are frequently associated with headaches in other populations [4, 20, 21], several factors may account for the lower reporting in this cohort. First, under-reporting of mild or non-disruptive headaches is likely, particularly in settings where patients prioritize more distressing psychological or somatic symptoms during clinical encounters [1, 18]. Cultural norms in Afghanistan may also contribute to under-expression of pain, as headache is often perceived as a routine or insignificant complaint and may therefore not be spontaneously reported unless severe. Second, somatic presentations of anxiety and depression vary across cultures; Afghan patients commonly express distress through fatigue, sleep problems, generalized body discomfort, or cardiopulmonary symptoms rather than localized pain such as headache [1, 6, 18]. Third, recall bias may have occurred because diagnostic interviews for non-psychotic disorders focused primarily on emotional and behavioral symptoms, which might have reduced the likelihood of detailed headache reporting. These contextual and methodological elements suggest that the findings do not contradict established clinical patterns but rather reflect how psychological distress is expressed and reported within this specific population.

Bivariate analysis elucidated several significant associations. Sleep disturbances were markedly higher in older patients (≥ 36 years, 79.9%), females (75.7%), and hypertensive individuals (78.3%) (all *p* < 0.001). This reinforces the widely recognized link between age, sex, and hypertension with compromised sleep quality [2, 3]. Previous research highlights that poor sleep can exacerbate hypertensive symptoms, and hypertension itself can negatively affect sleep quality, creating a bidirectional relationship [2]. Similarly, hypertension was more prevalent in older individuals (≥ 36 years, 76.0%), widowed/divorced individuals (80.4%), rural residents (59.3%), illiterate individuals (62.0%), and smokers (82.5%). These factors are well-established risk factors for hypertension, reflecting complex interactions between lifestyle, socioeconomic status, and health [8].

Multivariate regression analysis further refined these relationships, identifying older age (AOR = 3.680, *p* < 0.001) and widow status (AOR = 12.631, *p* < 0.001) as independent predictors for hypertension. The particularly strong association with widow status (AOR = 12.631) suggests that the psychosocial stress and socioeconomic challenges associated with widowhood in this cultural context might contribute to hypertension risk. Females had higher odds of both sleep disturbances (AOR = 1.614, *p* = 0.014) and headaches (AOR = 1.546, *p* = 0.014), corroborating previous findings on sex-based differences in the prevalence of these conditions [8, 23]. Notably, widowed individuals exhibited higher odds across all three conditions: sleep disturbances (AOR = 6.823), hypertension (AOR = 12.631), and headaches (AOR = 4.491) (all *p* < 0.001). The strong independent association between widowhood and poor sleep quality, hypertension, and headache likely reflects multiple underlying psychosocial and socioeconomic mechanisms. In Afghanistan, widowhood is commonly accompanied by profound bereavement stress, financial instability, reduced social support, and increased caregiving burdens, all of which have well-established effects on sleep dysregulation, autonomic arousal, and cardiovascular risk. Chronic psychosocial stress activates the hypothalamic–pituitary–adrenal (HPA) axis and sympathetic nervous system, contributing to hypertension and stress-related headache disorders. Furthermore, widowed women in Afghanistan frequently assume sole household responsibilities under conditions of economic insecurity, compounding the physiological and emotional strain. These combined gendered and cultural stressors offer a plausible explanation for the markedly elevated AORs observed for widowed participants.

Given the cross-sectional design, the directionality of the observed associations cannot be definitively established. Reverse causality is plausible—for example, chronic hypertension or recurrent headaches may themselves impair sleep quality, or poor sleep may exacerbate perceptions of pain and elevate blood pressure. Similarly, unmeasured confounders such as BMI, diabetes, caffeine intake, psychotropic medication use, trauma exposure, and chronic pain conditions could contribute residual confounding despite adjustment for key sociodemographic variables. Therefore, the findings should be interpreted as associations rather than causal relationships, underscoring the need for longitudinal designs to better elucidate temporal pathways.

An important finding is that education level and economic status—despite showing strong associations in the bivariate analyses—did not remain significant in the multivariate models. This apparent disparity likely reflects the combined confounding influence of age and widowhood, both of which demonstrated exceptionally strong independent effects on sleep disturbances, hypertension, and headaches. Widowhood alone showed adjusted odds ratios ranging from 4.49 to 12.63 across outcomes, suggesting that the psychosocial and economic vulnerabilities inherent to widowhood in Afghanistan may overshadow the more general socioeconomic variables of income and education. Moreover, the sample exhibited limited variability in socioeconomic status, with 93.1% earning less than $100 per month and 74.3% being illiterate. Such restricted heterogeneity reduces the model’s ability to detect the independent effects of these variables. Consequently, while socioeconomic disadvantage is a well-established determinant of poor health, in this cohort its statistical signal was likely overridden by the more dominant and culturally embedded stressors associated with widowhood and older age.

The findings highlight the significant burden of interconnected health issues, particularly in socioeconomically challenged populations like those in Afghanistan. The high prevalence of illiteracy and low income among the study participants points to potential barriers in health literacy and access to preventive care, which could contribute to the observed health outcomes [1, 8]. The study’s focus on non-psychotic patients is crucial as it allows for an understanding of these comorbidities outside the direct influence of severe mental illness, though conditions like anxiety and depression were considered. The use of PSQI for sleep quality assessment is a strength, as it is a widely validated instrument [17, 24].

A number of limitations should be considered when interpreting these findings. First, the cross-sectional design precludes any causal inference; the observed associations are correlational, and longitudinal studies are required to clarify temporal and potentially bidirectional pathways between sleep disturbances, hypertension, and headaches. Second, recruitment was restricted to a single regional hospital in Herat and relied on hospital-attending non-psychotic adults, which may introduce selection bias and limit external validity. Patients who seek care in psychiatry and internal medicine outpatient clinics may differ from community-dwelling adults or patients in other Afghan provinces in terms of symptom severity, comorbidity profiles, and help-seeking behavior. Third, sleep quality was assessed using a self-report instrument (PSQI), which is subject to recall and reporting biases and was not corroborated with objective measures such as actigraphy or polysomnography. Fourth, although non-psychotic diagnoses were made by psychiatry specialists using DSM-5–informed clinical interviews, structured diagnostic instruments (e.g., SCID-5) were not employed, and headache subtypes were classified using clinical judgment without a formal ICHD-3 checklist. These factors may introduce diagnostic misclassification and reduce comparability with studies using fully standardized assessments. Fifth, important lifestyle and clinical covariates—including BMI, diabetes, dyslipidemia, level of physical activity, caffeine and tea consumption, opioid or alcohol use, and detailed psychotropic and antihypertensive medication regimens—were not collected, leaving scope for residual confounding. Finally, some subgroups, particularly current smokers and those in higher-income categories, were small, which may have led to unstable estimates and wide confidence intervals for these strata. Taken together, these limitations indicate that the findings are most generalizable to non-psychotic adult outpatients attending psychiatry and internal medicine clinics at Herat Regional Hospital, rather than to the Afghan adult population as a whole.

Future research could explore the specific mechanisms underlying the observed associations, particularly the strong link between widow status and increased odds of all three conditions. Investigating the impact of culturally sensitive interventions aimed at improving sleep quality and managing hypertension and headaches in vulnerable populations like widows in Afghanistan could also be beneficial. The high prevalence of sleep disturbances also suggests a need for increased awareness and accessible interventions for sleep hygiene and disorders, which may indirectly improve headache and hypertension management [3, 23]. Given the impact of these conditions on quality of life, as evidenced by previous research, addressing these interconnected health issues is paramount for improving overall patient well-being in Afghanistan [1].

## Conclusion

This study demonstrates a high prevalence of sleep disturbances, hypertension, and headaches among non-psychotic Afghan patients, with interrelationships between these conditions. Older age, female gender, widowhood, and smoking emerged as key predictors, with widowed individuals displaying the highest vulnerability across all three outcomes. These findings underscore the need for targeted, culturally sensitive interventions to address psychosocial stressors, improve sleep quality, and manage hypertension and headache disorders in high-risk groups, particularly widows and socioeconomically disadvantaged populations. Public health strategies should focus on enhancing health literacy, improving access to preventive care, and developing community-based support programs to mitigate the burden of these interconnected health issues.

## Data Availability

The datasets during and/or analyzed during the current study are available from the corresponding author on reasonable request.
